# High Sensitivity, Rapid Detection of Virus in High Traffic Environments

**DOI:** 10.3389/fbioe.2022.877603

**Published:** 2022-03-24

**Authors:** Lauren Waller, Zhilin Guo, Rui Tang, Zunming Zhang, Edward Wang, Jarred Yasuhara-Bell, Louise Laurent, Yu-Hwa Lo

**Affiliations:** ^1^ Department of Bioengineering, University of California, San Diego, San Diego, CA, United States; ^2^ Department of Mechanical and Aerospace Engineering, University of California, San Diego, San Diego, CA, United States; ^3^ Department of Electrical and Computer Engineering, University of California, San Diego, San Diego, CA, United States; ^4^ Independent Consultant, San Diego, CA, United States; ^5^ Department of Obstetrics and Gynecology and Reproductive Sciences, University of California, San Diego, San Diego, CA, United States

**Keywords:** COVID19, SARS-cov-2, lateral flow assay, RT-LAMP, rapid diagnostics, point of care diagnostics, multiplexed loop mediated isothermal amplification

## Abstract

The global pandemic caused by the SARS-CoV-2 virus has underscored the need for rapid, simple, scalable, and high-throughput multiplex diagnostics in non-laboratory settings. Here we demonstrate a multiplex reverse-transcription loop-mediated isothermal amplification (RT-LAMP) coupled with a gold nanoparticle-based lateral flow immunoassay (LFIA) capable of detecting up to three unique viral gene targets in 15 min. RT-LAMP primers associated with three separate gene targets from the SARS-CoV-2 virus (Orf1ab, Envelope, and Nucleocapsid) were added to a one-pot mix. A colorimetric change from red to yellow occurs in the presence of a positive sample. Positive samples are run through a LFIA to achieve specificity on a multiplex three-test line paper assay. Positive results are indicated by a characteristic crimson line. The device is almost fully automated and is deployable in any community setting with a power source.

## Introduction

In late December 2019, a major outbreak of a novel coronavirus of zoonotic origin broke out in Wuhan, Hubei province, China (Wang et al., 2020). The rampant global spread of COVID-19 has shocked and forever changed the world, with 271,376,643 confirmed cases and 5,324,969 deaths globally as of 16 December 2021 (World Health Organisation, 2021) [“WHO Coronavirus (COVID-19) Dashboard” 2021].

Socioeconomic expansion and increased travel in this modern age have become associated with a rise in the emergence of novel viruses, with notable examples including the 2003 severe acute respiratory syndrome coronavirus (SARS) outbreak, the 2009 swine flu pandemic, the 2012 Middle East respiratory syndrome coronavirus outbreak (MERS), the 2013-2016 Ebola epidemic, and the 2015 Zika virus epidemic (Baker et al., 2021). With modern transportation capable of moving a person with a contagious illness to nearly any location in the world in under 24 h, the potential for highly contagious new outbreaks poses an incredible global health risk.

Novel respiratory pathogens such as SARS-CoV-2 have an established precedent of elevated transmissibility *via* high-traffic hubs of national or international travel such as airports, train stations, and cruise terminals (Pavia 2007) (Luo et al., 2021). Fueled by its high reproduction number, asymptomatic carriers, superspreading events, increasing socio economic globalization, and an immunologically naive population, COVID-19 has wreaked havoc across the world (Hu et al., 2020).

More than ever before, rapid diagnostic tests have become a fundamental component of outbreak mitigation, with crucial roles ranging from initial detection and quarantining procedures to population-level monitoring (Oeschger et al., 2021). Although a number of diagnostic technologies exist, RT-qPCR is commonly considered the gold standard and is consequently the most widely adopted diagnostic method in test centers and hospitals. The technique consists of three steps: collection of nasopharyngeal or oropharyngeal swabs from patients; total RNA extraction or isolation; and specific detection of target viral genome sequences *via* RT-qPCR. In RT-qPCR, the viral RNA is transcribed into cDNA by reverse transcriptase. The cDNA is the template for qPCR. Amplification products are quantified by the addition of a fluorescent label during replication. As the reaction proceeds, the fluorescence signal is directly proportional to DNA concentration and the linear correlation can be used to determine the amount of template at the start of the reaction (Kralik and Ricchi 2017). The inclusion of reverse transcription for the diagnosis of RNA viruses provides the necessary sensitivity and specificity for widespread use in a laboratory setting.

However, RT-qPCR is not sufficient for use in many high-traffic community settings for a number of reasons. This technique includes several sample handling steps; therefore, the sample-to-result procedure in a clinical diagnostic laboratory can take about 3–24 h or more, depending on the test center (Dao Thi et al., 2020). RT-qPCR also requires specialized lab equipment such as thermocyclers and fluorometers, and skilled operational personnel. These requirements are not easily implementable in community settings without significant equipment, training, and staffing costs, especially in underdeveloped and remote areas.

Isothermal amplification techniques are a popular solution for bringing diagnostic testing to non-laboratory environments due to its impressive sensitivity and ability to be performed with minimal resources (Sahoo et al., 2016). While a large number of isothermal amplification methods exist, loop-mediated isothermal amplification (LAMP) is amongst the most popular. LAMP enables nucleic acid amplification in 10–40 min using 4 to 6 primers and a polymerase with chain displacing ability at a constant temperature of around 65°C. In the case of reverse transcription LAMP (RT-LAMP), the process is initiated by reverse transcription from the backward inner primer (BIP) by binding onto the 3’ end. Concurrently, a new cDNA strand is created by binding of the B3 primer to the template aided by a strand displacing DNA polymerase. Loops at the 3’ end are created as the single stranded copy binds to itself. Then, the forward inner primer (FIP) binds to the 5’ end and aided by DNA polymerase, synthesizes a complementary strand. The F3 primer with DNA polymerase binds to the complementary strand and generates a new double stranded DNA while displacing the previous single strand. The amplification products are an amalgamation of target RNA and primers. The single stranded DNA forms dumbbell-like structures as the ends fold and bind to themselves. Exponential amplification of the dumbbell-like structure is then initiated. The addition of loop primers greatly speeds up the reaction (Huang et al., 2020) (Mautner et al., 2020) (Thompson and Lei, 2020).

The detection of target amplification products in RT-LAMP assays is frequently colorimetric. One common method is to use a pH indicator (e.g., phenol red) and execute the reaction in a weakly buffered environment. The lowering of the pH as the reaction proceeds results in a visible color change from red to yellow (Dao Thi et al., 2020). With pre-mixed reverse transcription and colorimetric LAMP reagents widely available (e.g., WarmStart Colorimetric LAMP 2X Master Mix, New England Biolabs, MA, United States), RT-LAMP is an appealing technique for deployment in high-traffic community settings. The colorimetric detection can be accelerated by adding chemicals (e.g., guanidine hydrochloride) and primers associated with additional target gene segments from the same virus. In the same spirit, primers for multiple diseases can be multiplexed to yield a single positive or negative in a one-pot mixture.

To achieve specificity of specific disease targets within this one-pot mixture, RT-LAMP can be coupled with a lateral flow immunoassay (LFIA). LFIAs offer an enticing solution to point-of-care diagnostics due to their low cost, high specificity, and simplicity of use (Koczula and Gallotta 2016) (Zhu et al., 2020) (Chen et al., 2021) (Zhang et al., 2021). Lateral flow assays are a paper-based platform that allows a sample to be placed on a test device and results to be displayed in about 10 min. The device usually consists of a sample pad, a conjugate pad, a nitrocellulose membrane, and an absorbent pad. The sample is mixed with running buffers and placed onto the sample pad. The conjugate pad usually contains reporter particles such as functionalized gold nanoparticles. If a positive sample is present, the reporter particles will bind to the analyte. Striped lines of antibody on the nitrocellulose membrane capture the analyte, and a control line captures extraneous reporter particles to validate the test. Positive results for an analyte will appear as a red line.

Here we present a high-throughput, rapid diagnostic machine that can be employed in almost any high-traffic public setting for the rapid detection of up to three gene targets, and the method can be readily extended to more targets from different organisms. The system uses RT-LAMP to isothermally amplify target sequences and provide a rapid yes or no diagnostic response based on the colorimetric change from red to yellow. Then, the RT-LAMP product is run through a lateral flow immunoassay to provide specificity. Here we use three gene targets of SARS-CoV-2 (Orf1ab, N, and E); however, the diagnostic ability can be more broadly applied by substituting the appropriate primers for other viral gene targets.

## Materials and Methods

### Automated System Architecture

A customized machine is utilized to automate all steps beyond the loading of patient samples. The machine leverages an existing pipetting robot (OT2-Pipettes, Opentrons, Long Island City, NY, United States) for precision liquid transfer. Customized 96-well plate holders and cassette holders driven by stepper motors are used to convey 96-well plates and LFIA cassettes into and out from the machine. Position sensors are installed to ensure precision and repeatability. A customized heating unit is installed under the 96-well plate to enable LAMP reaction heating. The colorimetric reaction readouts are detected using an overview camera (Flea3 USB3, Model: FL3-U3-13E4C-C, Teledyne FLIR LLC). A process chiller unit (Temperature Module, Opentrons) is adopted for liquid storage (e.g., LAMP reagents).

The machine is controlled by a desktop computer and a two-level user interface. The first level user interface opens to the system developers, which includes the system installation and calibration access. After initial installation and calibration, the second level user interface is opened to system operators for information loading and result display. Given the simplicity of the graphical user interface (GUI), system operators can be trained in minutes.

### System Automation and Overarching Workflow

**FIGURE 1:**
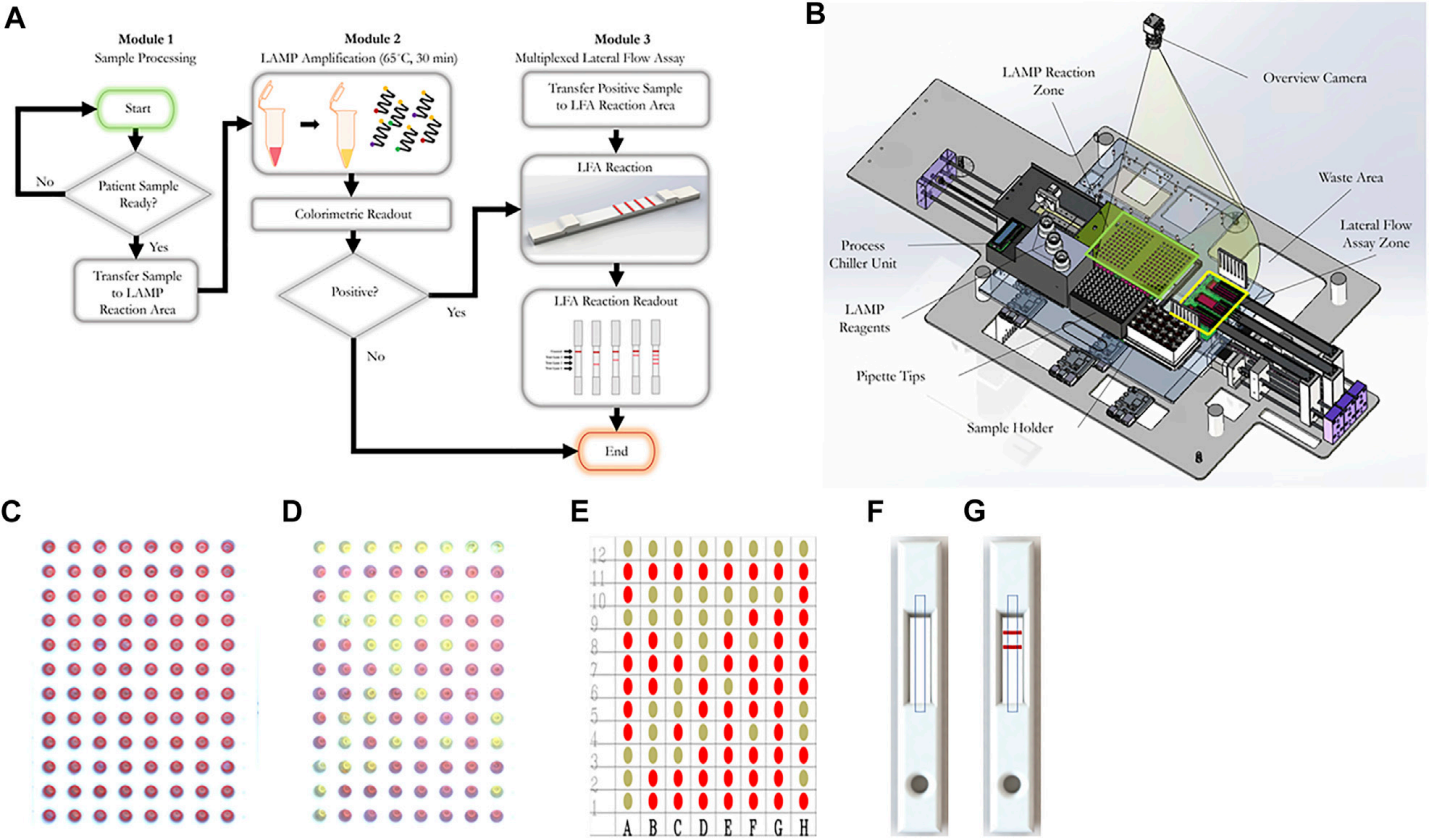
The workflow of our RT-LAMP-LFIA prototype system and the schematic of our automatic system. **(A)** Workflow schematic from initial patient sample placement to end. Samples undergo a colorimetric loop-mediated isothermal amplification reaction. Samples that are positive (as indicated by a colorimetric change from red to yellow) are transferred to a multiplexed lateral flow assay test strip, which can differentiate from up to three target sequences. **(B)** Machine schematic **(C)** Example 96-well plate image captured by the overview camera before RT-LAMP reaction. The red circle represents the region of interest (ROI) of each well. The decision matrix is determined by the color change within the red circles **(D)** Example 96-well plate image captured by the overview camera after RT-LAMP reaction. **(E)** Final decision matrix for RT-LAMP reaction readout **(F)** Example rendering of LFIA cassette image captured by the overview camera before LFIA. Dashed rectangles represent ROIs of the LFIA cassettes. **(G)** Example rendering of LFIA cassette image captured by the overview camera after LFIA.

Nasopharyngeal swabs are collected from patients using a nasal swab and stored in iSwab Microbiome-EL (Extraction-Less) buffer (MAWI DNA Technologies, Hayward, CA, United States). Collected patient samples are loaded into the sample holder by a human operator (Figure 1B). All reagents are stored on top of the process chiller unit. Upon initialization, 96-well plates and cassettes filled with LFIA are conveyed into the LAMP reaction zone and the lateral flow assay zone, respectively. After the system operator enters the sample information (e.g., number of samples, sample identification numbers), the machine first loads the pre-mixed LAMP reaction concoction (Table 1, Table 2) into a new 96-well plate. Next, the machine pipettes 2 µl of each patient sample into a single well in the 96-well plate. Pipette tips are changed after each sample to prevent cross-contamination of samples. Once all samples are loaded, the heating unit under the LAMP reaction zone heats the plate up to 65°C. A system timer starts once it reaches 65°C. After a preset reaction time (15–30 min), the RT-LAMP reaction is complete and positive samples are clearly identifiable by a colorimetric change from red to yellow. The colorimetric change is detected and quantitatively measured by the overview camera (Flea3 USB3, Model: FL3-U3-13E4C-C, Teledyne FLIR LLC), and the process is complete for samples that meet the threshold for a negative result. For samples that meet the criteria for a positive result, 3 µl of the LAMP product is mixed with 40 µl of running buffer (1 × PBS, pH 7.4 with 2% Tween 20) and deposited onto a lateral flow immunoassay. A system timer begins, and the result is detected and quantified after a predetermined amount of time. On the lateral flow immunoassay, positive results for each specific viral gene target will appear as a characteristic crimson line. A fourth crimson line acts as a control line and serves to validate the test. The positive readings which stand for the presence of COVID-19 RT-LAMP products are visible after 5 to 10 min using the LFIA. The colorimetric change of the LFIA is detected using the overview camera and then quantitatively measured on the computer. The final result for each sample is displayed on the user interface and a Portable Document Format (PDF) file including the sample information and detection results will be automatically stored in the local disk (or on the cloud if internet access is enabled). The system automatically displays a warning window on the user interface for 96-well plates and cassettes replenishment when needed (e.g., all the wells have been used).

**TABLE 1:** Concentrations of each oligonucleotide in the 10× primer mix.

Primer	10× Concentration (stock)	1 (µM) X Concentration (final)
F (µM)IP	16	1.6
BIP	16	1.6
F3	2	0.2
B3	2	0.2
LF	8	0.8
LB	8	0.8

**TABLE 2:** Concentrations of each reagent in the RT-LAMP assay.

Reagents	Volume (µl)
WarmStart Colorimetric LAMP 2X Master Mix	10
LAMP Primer Mix (10X)	2
Guanidine Hydrochloride	2
Nasopharyngeal Swab Sample	2
dH_2_O	4
Total Volume	20

### Quantification of the RT-LAMP Reaction and LFIA Readout

For the RT-LAMP reaction readout, after loading the new 96-well plates, the system first identifies the region of interest (ROI) of all wells. The RT-LAMP colorimetric readout is determined based on the color change within the ROI of each well. Two images are captured, one prior to the RT-LAMP reaction and one after the RT-LAMP reaction. YUV color space is used to compare the difference between two images. Wells that exceed the threshold are considered positive samples. Figures 1B,C,D, show an example image captured before and after the RT-LAMP reaction, respectively. The RT-LAMP reaction detection result is shown in Figure 1E.

For the LFIA readout, after loading the LFIA cassettes, the system will first identify the ROI of each LFIA cassette. The LFIA colorimetric readout is determined by the appearance or lack thereof of a crimson line within the ROI. Two images are captured, one prior to the LFIA reaction and one after the LFIA reaction. For each image, mean intensity along the width direction is obtained, which compresses the ROIs into two decision lines. YUV color space is used to compare the color difference between two lines. The final LFIA readout is obtained from the peak detection result from the YUV color space difference. Figures 1D,F,G, show an example rendered image captured before and after the LFIA reaction, respectively.

### RT-LAMP Primer Design and Viral Targets Information

RT-LAMP primers against Nucleocapsid (N-2), Envelope (E-2), and ORFla-1 genes (Table 3) were designed according to previously published specifications (Zhang et al., 2020) and were synthesized by Integrated DNA Technologies (IDT, San Diego, CA, United States). The sequence of primers and concentrations of each oligonucleotide in the 10x primer mix are shown in Tables 1, 2. The 10× primer mix was prepared by dissolving the oligonucleotides in nuclease-free water. It was stored at −20°C before use.

**TABLE 3:** Primer sequences against Nucleocapsid (N-2), Envelope (E-2), and Orf1a-1 genes of the SARS-CoV-2 virus.

Gene target	Primer	Name	Tag	Sequence
N2	FIP	N2 FIP	5’-FITC	TGC​GGC​CAA​TGT​TTG​TAA​TCA​GCC​AAG​GAA​ATT​TTG​GGG​AC
	BIP	N2 BIP	—	CGC​ATT​GGC​ATG​GAA​GTC​ACT​TTG​ATG​GCA​CCT​GTG​TAG
	F3	N2 F3	—	AACACAAGCTTTCGGCAG
	B3	N2 B3	—	GAA​ATT​TGG​ATC​TTT​GTC​ATC​C
	LF	N2 LF	—	TTC​CTT​GTC​TGA​TTA​GTT​C
	LB	N2 LB	5’-Biotin	ACCTTCGGGAACGTGGTT
Orf1a	FIP	Orf1a FIP	5’-DigN	GAG​GGA​CAA​GGA​CAC​CAA​GTG​TAT​GGT​TGA​GCT​GGT​AGC​AGA
	BIP	Orf1a BIP	—	CCA​GTG​GCT​TAC​CGC​AAG​GTT​TTA​GAT​CGG​CGC​CGT​AAC
	F3	Orf1a F3	—	CTG​CAC​CTC​ATG​GTC​ATG​TT
	B3	Orf1a B3	—	AGC​TCG​TCG​CCT​AAG​TCA​A
	LF	Orf1a LF	—	CCG​TAC​TGA​ATG​CCT​TCG​AGT
	LB	Orf1a LB	5’-Biotin	TTC​GTA​AGA​ACG​GTA​ATA​AAG​GAG​C
E2	FIP	E2 FIP	—	ACC​TGT​CTC​TTC​CGA​AAC​GAA​TTT​GTA​AGC​ACA​AGC​TGA​TG
	BIP	E2 BIP	—	CTA​GCC​ATC​CTT​ACT​GCG​CTA​CTC​ACG​TTA​ACA​ATA​TTG​CA
	F3	E2 F3	—	CCGACGACGACTACTAGC
	B3	E2 B3	—	AGA​GTA​AAC​GTA​AAA​AGA​AGG​TT
	LF	E2 LF	5’-TexRed	TCG​ATT​GTG​TGC​GTA​CTG​C
	LB	E2 LB	5’-Biotin	TGA​GTA​CAT​AAG​TTC​GTA​C

The RT-LAMP primer sets corresponding to the N-2, E-2, and ORF1a-1 genes were each designed to incorporate a unique tag onto each amplified RT-LAMP product. Texas Red was labeled on the 5’ end of the E-2 LF primer, FITC was labeled on the 5’ end of the N-2 FIP primer, and Digoxigenin was labeled on the 5’ end of the ORF1a-1 FIP primers. Biotin was labeled on the 5’ end of the LB primers for all three genes. Accordingly, Anti-Texas Red, Anti-Fluorescein, and Anti-Digoxigenin antibodies were striped onto the nitrocellulose membrane of the LFIA as test lines, while the biotin-BSA was used as the control line. Leveraging the natural affinity of streptavidin to biotin, which is one of the strongest non-covalent interactions in nature, streptavidin gold nanoparticles (Streptavidin, 40 nm 10 OD Colloidal Gold, Attogene, TX, United States) served as the reporter particle. Therefore, the RT-LAMP products for each gene only bind to one test line, enabling the detection of up to three distinct viral gene target detections on a single LFIA. Because in this instance all three gene targets were from RNA from the same virus and thus competition between templates can occur, gene blocks (sub-sequences of the entire SARS-CoV-2 RNA template) are used to validate the test. In the clinical and contrived viral tests, competition between primer sets can obscure some gene sequences on the LFIA; however, the RT-LAMP colorimetric change from red to yellow will be unaffected.

The SARS-CoV-2 RNA (VR-3280SD) was purchased from the American Type Culture Collection (ATCC, Manassas, Virginia, United States). The COVID-19 RNA was diluted as necessary by nuclease-free water. The Nucleocapsid (N), Envelope (E), and ORF1ab gene blocks were synthesized by Integrated DNA Technologies (IDT, IA, United States), with sequences shown in Table 4.

**TABLE 4:** Sequences of N, E, and Orf1ab gene blocks.

Gene blocks	Sequence
N gene (229 nt)	AAC​ACA​AGC​TTT​CGG​CAG​ACG​TGG​TCC​AGA​ACA​AAC​CCA​AGG​AAA​TTT​TGG​GGA​CCA​GGA​ACT​AAT​CAG​ACA​AGG​AAC​TGA​TTA​CAA​ACA​TTG​GCC​GCA​AAT​TGC​ACA​ATT​TGC​CCC​CAG​CGC​TTC​AGC​GTT​CTT​CGG​AAT​GTC​GCG​CAT​TGG​CAT​GGA​AGT​CAC​ACC​TTC​GGG​AAC​GTG​GTT​GAC​CTA​CAC​AGG​TGC​CAT​CAA​ATT​GGA​TGA​CAA​AGA​TCC​AAA​TTT​C
E gene (234 nt)	CCG​ACG​ACG​ACT​ACT​AGC​GTG​CCT​TTG​TAA​GCA​CAA​GCT​GAT​GAG​TAC​GAA​CTT​ATG​TAC​TCA​TTC​GTT​TCG​GAA​GAG​ACA​GGT​ACG​TTA​ATA​GTT​AAT​AGC​GTA​CTT​CTT​TTT​CTT​GCT​TTC​GTG​GTA​TTC​TTG​CTA​GTT​ACA​CTA​GCC​ATC​CTT​ACT​GCG​CTT​CGA​TTG​TGT​GCG​TAC​TGC​TGC​AAT​ATT​GTT​AAC​GTG​AGT​CTT​GTA​AAA​CCT​TCT​TTT​TAC​GTT​TAC​TCT
Orf1ab gene (289 nt)	TCC​AGA​TGA​GGA​TGA​AGA​AGA​AGG​TGA​TTG​TGA​AGA​AGA​AGA​GTT​TGA​GCC​ATC​AAC​TCA​ATA​TGA​GTA​TGG​TAC​TGA​AGA​TGA​TTA​CCA​AGG​TAA​ACC​TTT​GGA​ATT​TGG​TGC​CAC​TTC​TGC​TGC​TCT​TCA​ACC​TGA​AGA​AGA​GCA​AGA​AGA​AGA​TTG​GTT​AGA​TGA​TGA​TAG​TCA​ACA​AAC​TGT​TGG​TCA​ACA​AGA​CGG​CAG​TGA​GGA​CAA​TCA​GAC​AAC​TAC​TAT​TCA​AAC​AAT​TGT​TGA​GGT​TCA​ACC​TCA​ATT​AGA​GAT​GGA​ACT​TAC​ACC​AGT​TGT​TCA​GAC​T

### Lateral Flow Immunoassay

**FIGURE 2:**
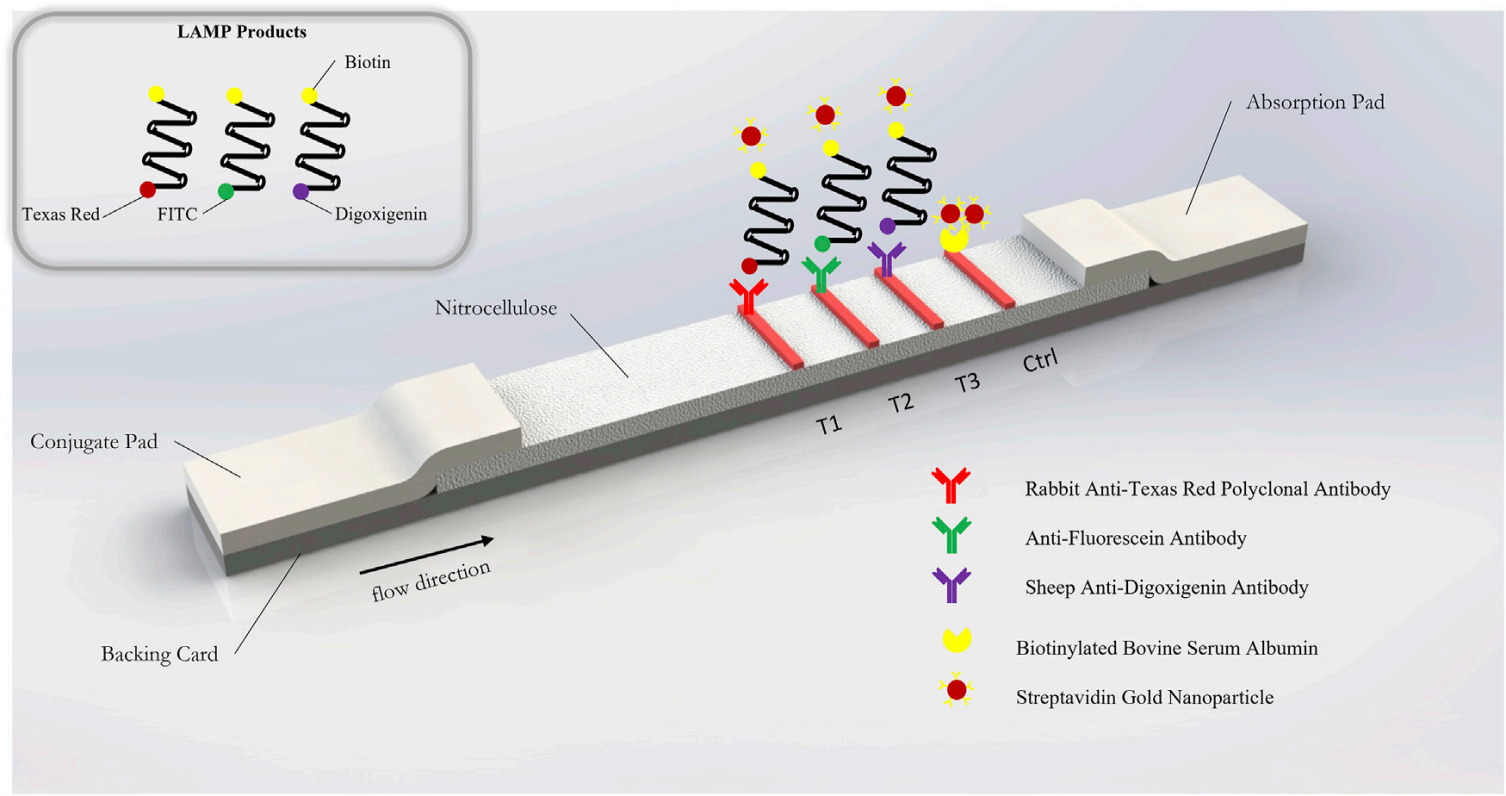
Schematic of gold nanoparticle based lateral flow immunosorbent assay for the detection of SARS-CoV-2 Orf1a, N2, and E2 target genes.

The lateral flow immunoassay (LFIA) (Figure 2) was comprised of a conjugate pad (GFCP203000 Glass Fiber Conjugate Pad Sheets, Millipore Sigma, St. Louis, MO, United States), a nitrocellulose membrane (Whatman FF120HP, Cytiva Life Sciences, Marlborough, MA, United States), an absorbent pad (Blotting Paper, Grade 222, Ahlstrom-Munksjö, Helsinki, Finland), and a backing card (DCN Diagnostics, Carlsbad, CA, United States). The dimensions of the conjugate pad were 2 cm × 30 cm; the dimensions of the nitrocellulose membrane were 2.5 cm × 30 cm; the dimensions of the absorbent pad were 2 cm × 30 cm); and the dimensions of the backing card were 6 cm × 30 cm. The conjugate pad was soaked in a blocking solution (1% bovine serum albumin + 0.05% Tween 20 + 0.2% sucrose, diluted in PBS) on a shaker at low speed for 10 min and the conjugate pads were dried on a drying rack overnight. Anti-texas red antibody (anti-TexR, 1 mg/ml, Cat. #A-6399, ThermoFisher Scientific, Waltham, MA, United States), rabbit anti-fluorescein antibody (anti-FITC, 1 mg/ml, Cat. # ab19491, Abcam, Cambridge, United Kingdom), sheep anti-digoxigenin antibody (anti-DigN, 2.5 mg/ml, Cat. #3210-0488, Bio-Rad, Hercules, CA, United States) and biotinylated bovine serum albumin (biotin-BSA, 3 mg/ml, Cat. #B-2007-10, Vector Labs, Burlingame, CA, United States) were stripped at the detection regions on the nitrocellulose using a lateral flow reagent dispenser (Claremont Biosolutions, Upland, CA, United States) at test line 1 (TL1), test line 2 (TL2), test line 3 (TL3) and control line (Ctrl), respectively, with each line separated by 5 mm. The membranes were then assembled together and the assembled LFIA were cut into 4-mm dipsticks. Then, 3 µl of streptavidin gold nanoparticles (Streptavidin, 40 nm 10 OD Colloidal Gold, Attogene, TX, United States) were immobilized at one end of the conjugate pad for each strip and dried in a vacuum. The dipsticks were packaged in a plastic cassette (DCNDx, Carlsbad, CA, United States) and stored in a 4°C fridge in a heat-sealed foil packet with a silica gel desiccant until use. The LFIAs were tested 3 months later and found to still be viable.

### Quantification of the RT-LAMP Reaction and LFIA Readout

The colorimetric change of the RT-LAMP reaction is quantitatively measured by the overview camera (Flea3 USB3, Model: FL3-U3-13E4C-C, Teledyne FLIR LLC). The prototype algorithm identifies the region of interest (ROI) of all RT-LAMP wells in the 96-well plate. The grayscale and red values of each well are evaluated inside the ROI. In each batch, at least three negative samples are present to serve as negative controls. The average value of the red/grayscale value of these negative controls are used to normalize the red/grayscale value for every well. Intuitively, a larger red/grayscale value means that the sample is more red, corresponding to a negative result of colorimetric RT-LAMP. A smaller red/grayscale value means that the sample is less red–and thus a colorimetric change from red to yellow has occurred. Accordingly, our algorithm assigns a cutoff value of > 0.98 for negative samples and < 0.98 for positive samples. Independently, the same algorithm was employed using the built-in tools of ImageJ for quantification for the experiments denoted in the Results section.

### Clinical Sample Handling and RT-qPCR

Clinical samples were obtained from the UCSD COVID-19 Research Biobank (University of California, San Diego, CA, United States). Nasopharyngeal swabs from patients both positive and negative for the SARS-CoV-2 virus were collected in iSwab Microbiome-EL (Extraction-Less) buffer. Collected samples were aliquoted into freezer tubes in a BSL2 + hood and were stored at −80°C prior to use. RT-qPCR was performed on all samples. Cq values are noted in Table 5.

**TABLE 5:** Cq values obtained from RT-QPCR for clinical nasopharyngeal specimens.

Sample no	Test result	Cq value
1-30	Negative	Undetectable
31	Positive	27.514
32	Positive	33.405
33	Positive	25.0205
34	Positive	27.708
35	Positive	28.526
36	Positive	32.840
37	Positive	27.422
38	Positive	32.859
39	Positive	30.810
40	Positive	29.406
41	Positive	27.204
42	Positive	32.703
43	Positive	35.175
44	Positive	25.782
45	Positive	21.299
46	Positive	34.516
47	Positive	26.791
48	Positive	31.589
49	Positive	27.127
50	Positive	25.745
51	Positive	26.304
52	Positive	26.203
53	Positive	31.114
54	Positive	34.482
55	Positive	33.929
56	Positive	28.251
57	Positive	34.532
58	Positive	35.496
59	Positive	33.400
60	Positive	31.301

## Results

### Time-To-Result Test

**FIGURE 3:**
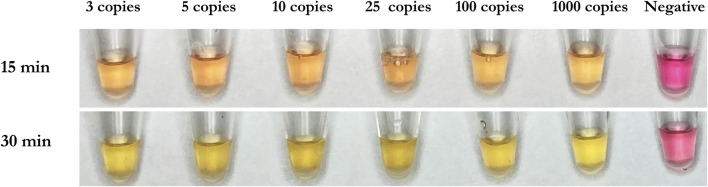
Demonstrative colorimetric loop-mediated isothermal reaction using primers as listed in Table 1. Viral RNA from SARS-CoV-2 (ATCC) were spiked in the amounts of 0, 3, 5, 10, 25, 100, and 1,000 RNA copies per microwell and reactions were imaged after 15 and 30 min, respectively.

WarmStart Colorimetric LAMP 2X Master Mix and guanidine hydrochloride solution (New England Biolabs, Ipswich, MA, United States) were used to detect the SARS-CoV-2 RNA template. The RNA template was diluted by nuclease-free water into samples with 3, 5, 10, 25, 50, 100, and 1,000 genome copies/ well. The three custom RT-LAMP primers against Nucleocapsid (N-2), Envelope (E-2), and ORFla-1 genes (Table 3) were mixed together. The colorimetric RT-LAMP reaction was conducted at 65°C and imaged after 15 and 30 min. The test demonstrated a sensitivity of 3 copies/well of SARS-CoV-2 RNA template after 15 min, as observed by the red to yellow color change, while the negative sample stayed red after the reaction (Figure 3). The 30 min time point was chosen for subsequent experiments to allow ample time for the reaction to proceed to completion.

### Multiplexed Gene Detection by Colorimetric RT-LAMP and LFIA

**FIGURE 4:**
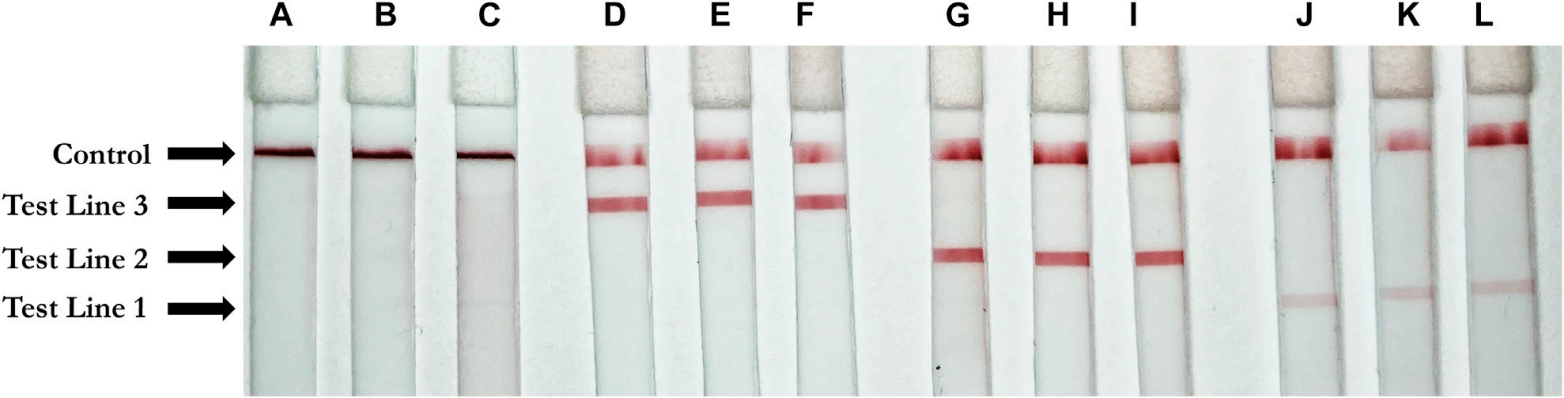
Positive test results for three unique gene blocks (subsections of SARS-CoV-2 viral genome). Test Line 1 (Texas Red Antibody, 1 mg/ml, Cat. #A-6399, ThermoFisher Scientific, Waltham, MA, United States); Test line 2 (Fluorescein antibody, 1 mg/ml, Cat. #ab19491, Abcam, Cambridge, United Kingdom); Test Line 3 (Digoxigenin antibody, 2.5 mg/ml, Cat. #3210-0488, Bio-Rad, Hercules, CA, United States); Control line (biotinylated bovine serum albumin, 3 mg/ml, Cat. #B-2007-10, Vector Labs, Burlingame, CA, United States) **(A–C)** Negative control. **(D–F)** Orf1a gene block (1 ng/μl) **(G–I)** N gene block (1 ng/μl) **(J–L)** E gene block (1 ng/μl).

Using each single gene block (N, E, and Orf1ab), RT-LAMP reactions were carried out in triplicate with all three primers present. Following the reaction, 3 µl of LAMP product was applied to the sample region of the LFIA with 40 µl of running buffer. As expected, only one positive line relative to the corresponding amplified RT-LAMP product was observed on each strip, confirming that in the presence of a single non-competing RNA template (gene block), the three mixed primer sets from Orf1a, E-2, and N-2 genes will produce a single positive result on the LFIA corresponding to their appropriate tag (Digoxigenin, Texas Red, or Fluorescein). The weaker signal for E2 is attributed to natural variation in antibody efficacy but is still clearly visible. The no template control showed no result at any test line. Accumulation of streptavidin AuNPs at the control line was observed in all cases. Results are depicted in Figure 4.

### Sensitivity of Colorimetric RT-LAMP and LFIA

**FIGURE 5:**
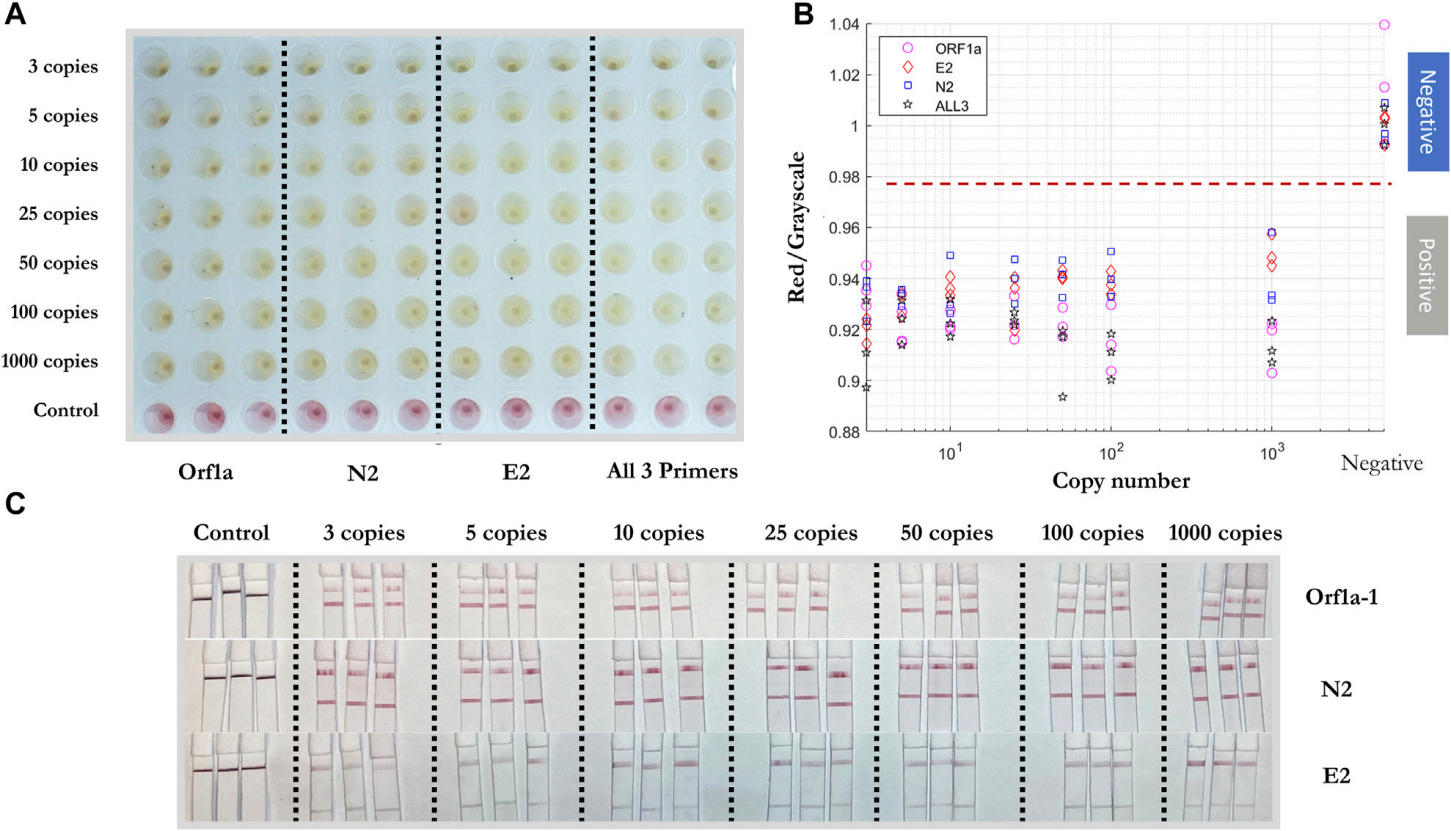
Sensitivity test of colorimetric RT-LAMP reaction and LFIA of artificial SARS-CoV-2 RNA template. **(A)** Positive/negative RT-LAMP products with ORF1a-1, N-2, E-2, and all three primer sets after the reaction, taken by overview camera. **(B)** Red/grayscale value vs. RNA copy numbers of both positive and negative samples. **(C)** LFIA read-outs for RT-LAMP products amplified for ORF1ab, N, and E gene blocks.

To test the sensitivity of the colorimetric RT-LAMP reaction of the SARS-CoV-2 RNA template, N-2, E-2, and ORF1a-1 primer sets were added individually and together in colorimetric RT-LAMP master mix solutions with 3, 5, 10, 25, 50, 100, and 1,000 RNA copies per well (Figure 5). All conditions were tested in triplicate. All positive samples turned yellow within 30 mins, while negative samples remained red. The results were quantified using red/grayscale values measured by ImageJ. Positive samples amplified by primer sets alone or together were distributed between 0.9 and 0.96. For negative samples, these values were around 1. For RT-LAMP products amplified by one set of primers, 3 µl of each amplification product was run on the LFIA. As expected, only one positive line relative to the appropriate amplified gene was observed on each strip for all positive samples.

**FIGURE 6:**
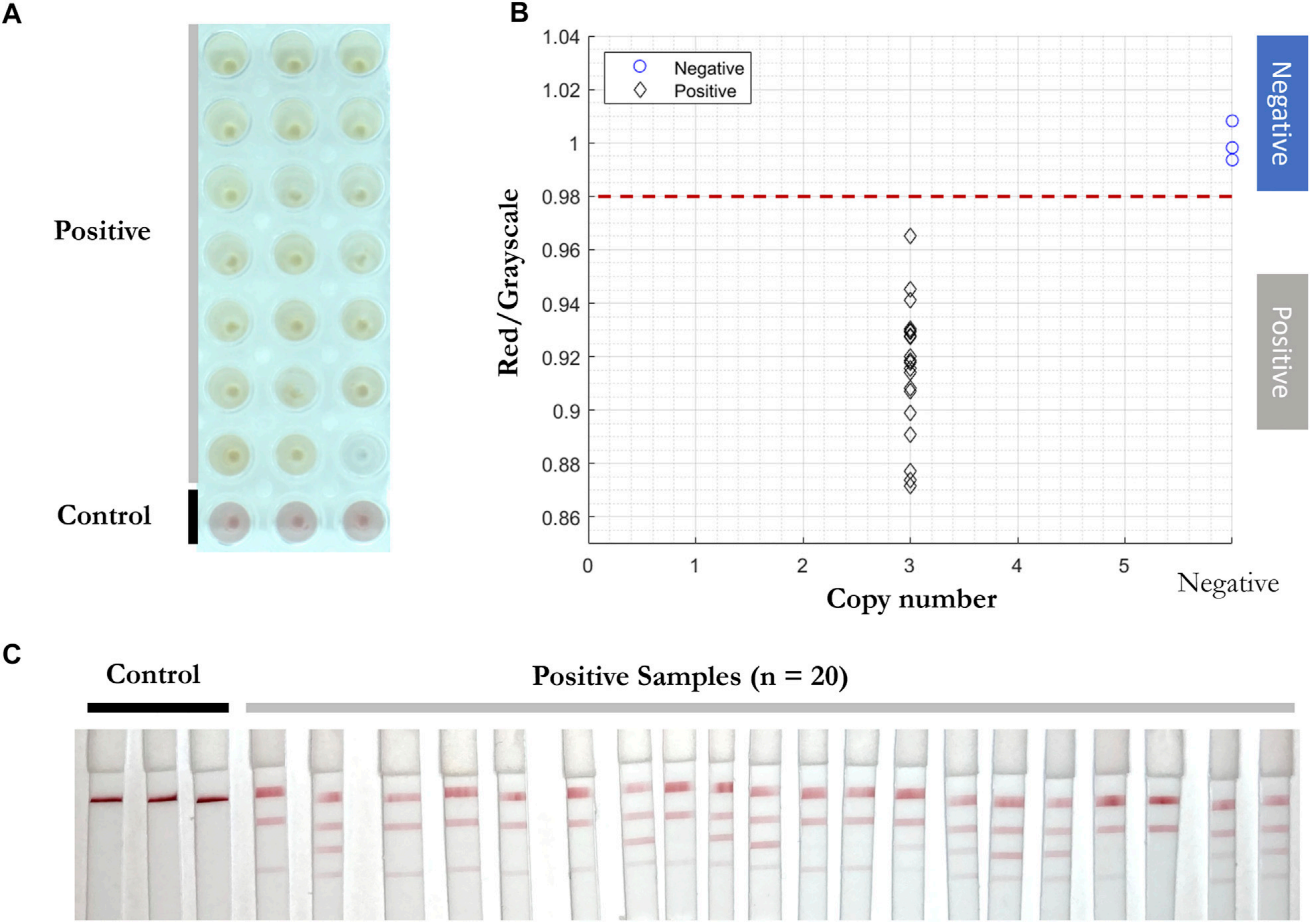
Reproducibility test of RT-LAMP-LFIA prototype system at the lower limit of detection. **(A)** RT-LAMP products from 3 initial template copies (*n* = 20) and negative control samples (*n* = 3) after the reaction, taken by overview camera. **(B)** Red/grayscale vs. copy numbers of both positive and negative samples. **(C)** LFIA read-outs for RT-LAMP products amplified for all three genes.

To test the reproducibility of the RT-LAMP-LFIA prototype system at the lower limit of detection, all three primers were added into the RT-LAMP master mix with 3 copies of SARS-CoV-2 RNA template for twenty replicates, with triplicates of negative controls. The experiment was carried out at 65°C for 30 min. A red to yellow color change was observed by the prototype camera from above. The red/grayscale values measured by ImageJ distributed below 0.98 for positive samples, while the values for negative control samples were around 1 (Figure 6).

As expected, 5 to 10 min after adding the RT-LAMP products and running buffer onto each conjugate pad of the LFIA, different positive readings were shown. In the presence of a single RNA target and three sets of primers, competition for a limited amount of template strands means that some reactions can overtake others. As a self-limiting reaction, the process stops when a certain amount of products form or the reaction reaches certain (acid) pH values. Therefore, the amount of N-2, E-2, and ORF1a-1 primer sets’ amplified products can vary and show different positive read-outs on the LFIA (Figure 6C).

### Validation of RT-LAMP and LFIA for Inactivated Whole Viral Particles

**FIGURE 7:**
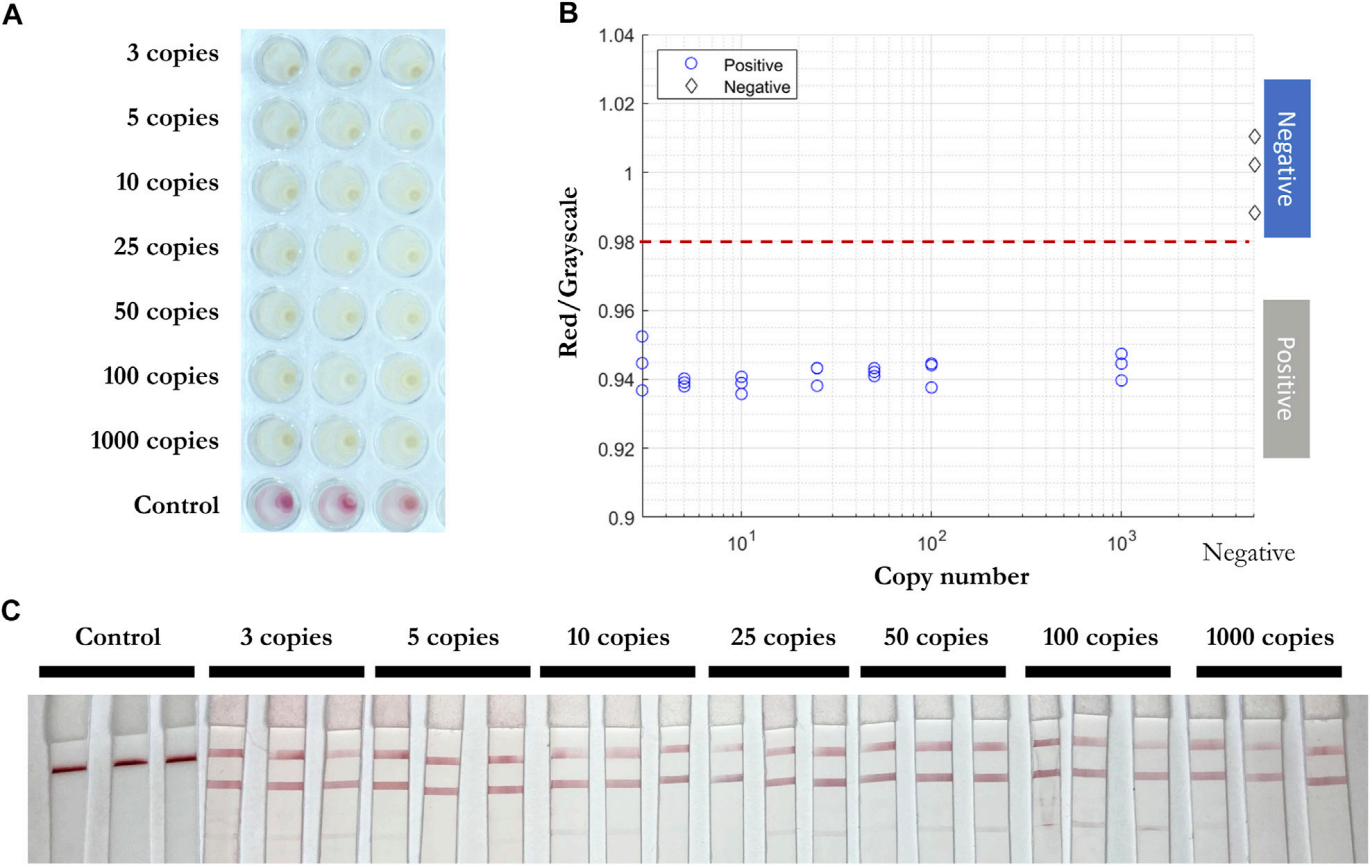
Direct RT-LAMP-LFIA testing of inactivated viral particles without prior RNA purification or extraction. **(A)** RT-LAMP products for inactivated viral particles (3, 5, 10, 25, 50, 100, and 1,000 copies) and negative control samples with three sets of primers, taken by overview camera. **(B)** Red/grayscale vs. copy numbers of both positive and negative samples. **(C)** LFIA read-outs for RT-LAMP products amplified for all three genes.

RT-qPCR (Smyrlaki et al., 2020) (Wee et al., 2020) and RT-LAMP assays (Lamb et al., 2020) (Rabe and Cepko 2020) are compatible with direct testing of nasopharyngeal and oropharyngeal swab specimens without an RNA purification or extraction step. To test the efficiency of RT-LAMP and LFIA, inactivated viral particles (ATCC, VR-1986HK) diluted into 3, 5, 10, 25, 50, 100, and 1,000 copies were added in the RT-LAMP master mix with the three gene primer sets (Figure 7). After 30 min at 65°C, a red to yellow color change was captured in wells with the inactivated viral particles by the prototype system camera. The red/grayscale value as measured by ImageJ for positive samples was distributed at around 0.94, while all the negative samples had red/grayscale values above 0.98. Next, 3 µl of each amplification product was mixed with 40 µl of running buffer before being deposited on the conjugate pad of a LFIA strip. Positive read-outs were mainly observed for ORF1a-1 gene amplified products (Figure 7C) as a result of primers competing during the self-limiting RT-LAMP reaction.

**FIGURE 8:**
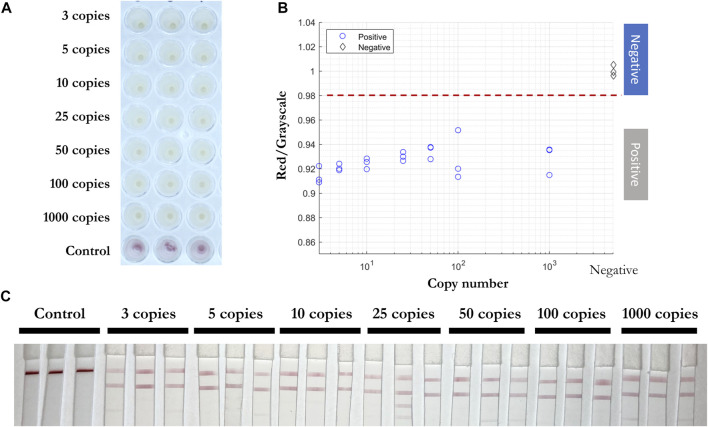
Direct RT-LAMP-LFIA testing of spiked inactivated viral particles in nasopharyngeal swab specimens without a prior RNA purification or extraction. **(A)** RT-LAMP products for inactivated viral particles spiked in nasopharyngeal swab specimens (3, 5, 10, 25, 50, 100, and 1,000 copies) and negative control samples with three sets of primers, taken by overview camera. **(B)** Red/grayscale vs. copy numbers of both positive and negative samples. **(C)** LFIA read-outs for RT-LAMP products amplified for all three genes.

Next, inactivated viral particles were spiked into unique negative patient swab samples preserved in iSwab Microbiome-EL (Extraction-Less) buffer to mimic patient-to-patient variation of conditions in amounts of 3, 5, 10, 25, 50, 100, and 1,000 copies per reaction. All samples were red prior to the RT-LAMP reaction, and positive samples were yellow after 30 min at 65°C. The red/grayscale values of positive samples were consistently below 0.96, while the negative samples were distributed around 1 (Figure 8). Due to primers competing during the self-limiting RT-LAMP reaction, positive read-outs on the LFIA were mainly observed for ORF1a-1 gene amplified products (Figure 8C).

### Validation of RT-LAMP and LFIA For Clinical Samples

**FIGURE 9:**
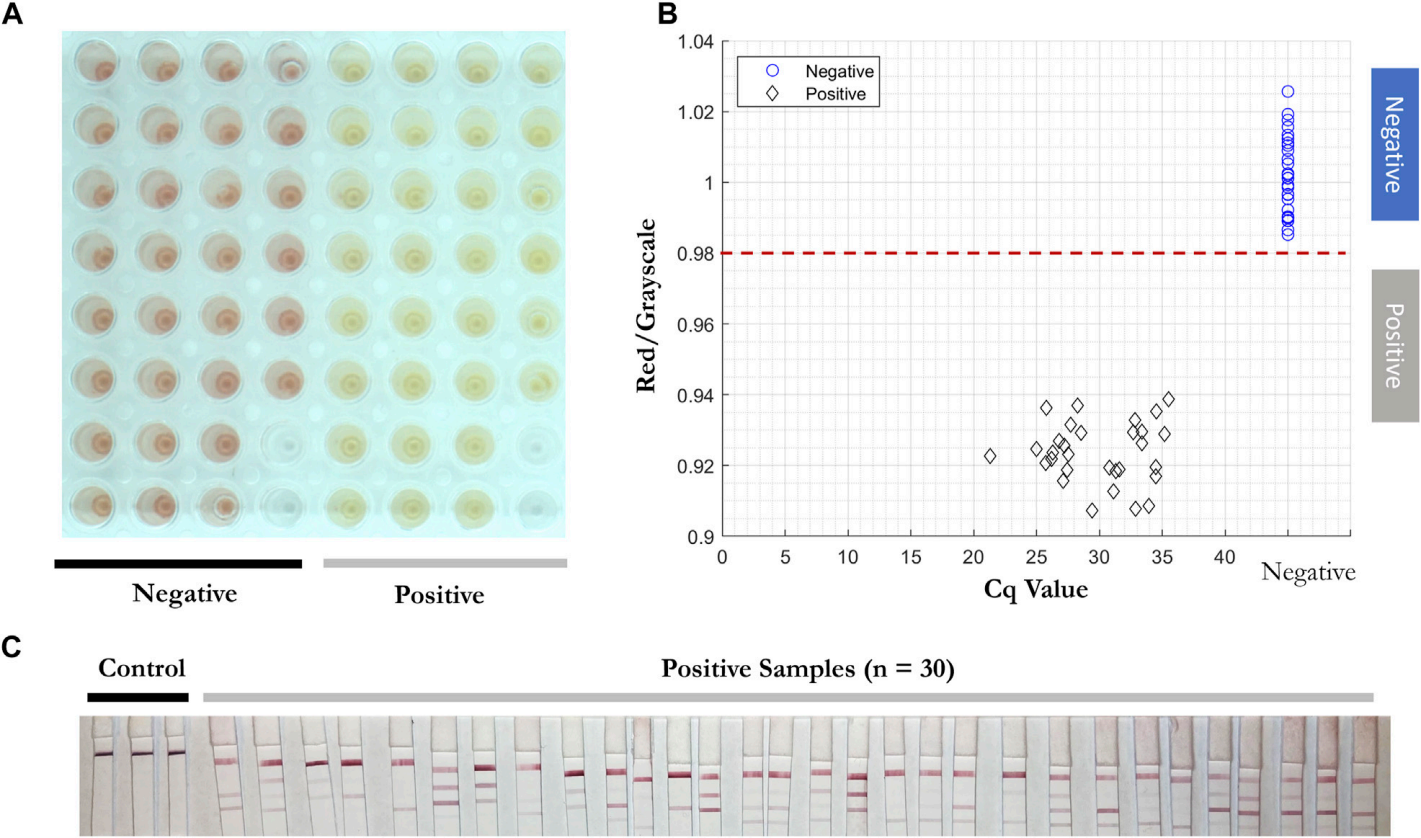
Direct RT-LAMP-LFIA testing of 30 positive and 30 negative clinical nasopharyngeal swab specimens without a prior RNA purification or extraction. **(A)** RT-LAMP products for 30 positive and 30 negative clinical samples amplified with three sets of primers, taken by overview camera. **(B)** Red/grayscale vs. copy numbers of both positive and negative samples. **(C)** LFIA read-outs for RT-LAMP products amplified for all three genes.

2 µl of solution from 30 positive and 30 negative clinical samples (Table 5) were added into RT-LAMP master mix with all three primer sets present. The positive samples turned yellow within 30 min at 65°C and had red/grayscale values between 0.9 and 0.94. The negative samples remained red with red/grayscale values above 0.98 as quantified by ImageJ (Figure 9). For positive samples, read-outs were observed for all samples on at least one test line on the LFIA (Figure 9C).

## Discussion

In this paper, we presented a semi-automated RT-LAMP-LFIA prototype system and evaluated its capability to test nasopharyngeal swab samples from patients without a prior RNA purification or extraction step. The system is almost fully automated and does not require trained personnel or specialized laboratory equipment such as thermocyclers. The simple design and automated sample-to-answer workflow indicate that our system is suitable for implementation in high-traffic community settings such as airports or event centers for rapid and easy to use point-of-care diagnostics.

We tested RT-LAMP primer sets for N, E, and ORF1ab genes of the SARS-CoV-2 virus and validated their results on the LFIA. Multiplexing of colorimetric RT-LAMP reactions in a single pot for the SARS-CoV-2 gene targets accelerated the reaction to yield results within 15 min. Moreover, the multiplexed read-out of up to 3 genes on a single LFIA can further distinguish multiple viral public health threats on the same platform - for example, SARS-CoV-2, Ebola, and yellow fever.

The main advantages of the RT-LAMP-LFIA method–its simplicity, low cost, high sensitivity, and high specificity–makes it ideal for use as a high-throughput diagnostic device in non-laboratory settings. To apply the method and system to detect multiple viruses or their variants, one can extend the primer set and divide the sample into a few LAMP reaction pots, each containing a chosen set of gene targets for specific organisms. Thus the overall technique and system reported here are scalable to protect the public from future outbreak and epidemic.

## Data Availability

The original contributions presented in the study are included in the article/Supplementary Material, further inquiries can be directed to the corresponding author.
