# Supplementary material for: High Sensitivity, Rapid Detection of Virus in High Traffic Environments
Source: Front Bioeng Biotechnol. 2022 Mar 24;10:877603. doi: 10.3389/fbioe.2022.877603 (PMC8989402; doi:10.3389/fbioe.2022.877603)
Supplement: Supplementary file 1 [file DataSheet1.PDF]

Supplementary Material

1.1 Supplementary Figures

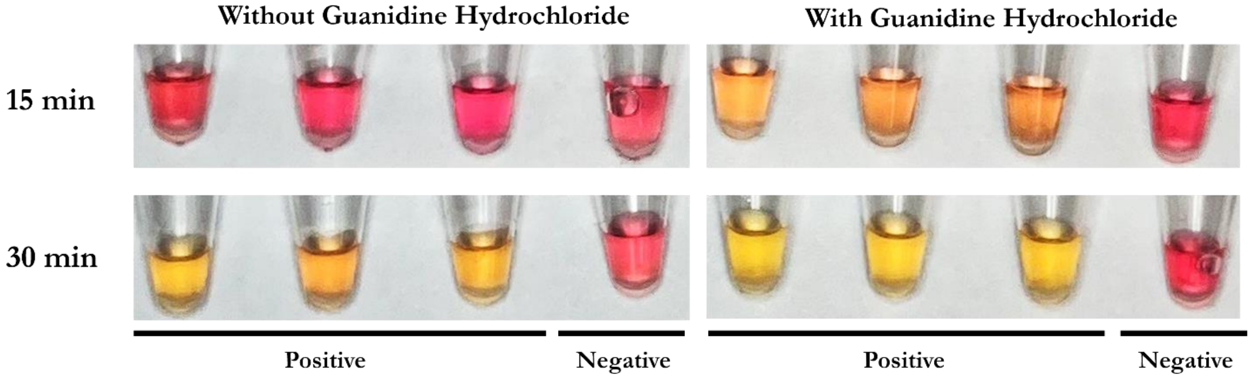

Supplementary Figure 1. Comparison of LAMP reaction times with and without guanidine hydrochloride. LAMP reactions with and without guanidine hydrochloride were heated in dry bath at 65˚C for 15 min and 30 min. Positive samples contained 1000 copies of SARS-Cov-2 RNA. Negative samples contained water.

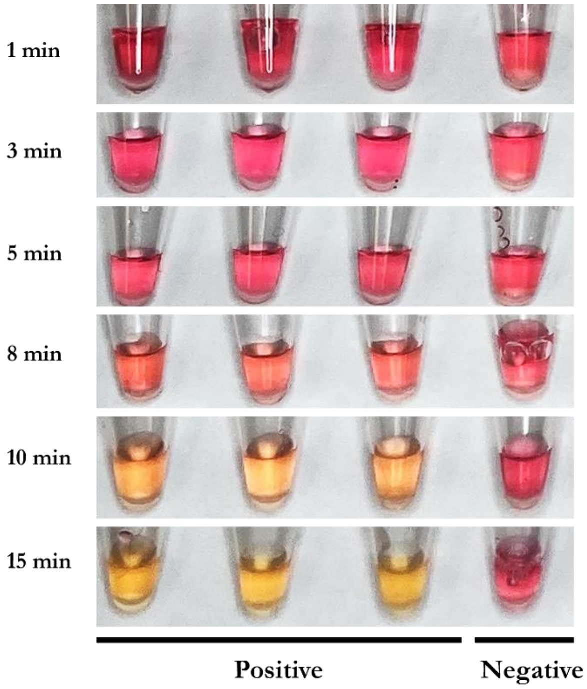

Supplementary Figure 2. LAMP reaction color change progression over time. LAMP samples with guanidine hydrochloride were heated in dry bath at 65˚C for 1, 3, 5, 8, 10 and 15 min, respectively. Positive samples contained 1000 copies of SARS-Cov-2 RNA. Negative samples contained water.
